# The predictive role of sickness absence spell durations in associations with inpatient- and specialized outpatient care among a population-based Swedish twin sample

**DOI:** 10.1186/s12913-021-06310-w

**Published:** 2021-04-07

**Authors:** Annina Ropponen, Mo Wang, Jurgita Narusyte, Sanna Kärkkäinen, Victoria Blom, Pia Svedberg

**Affiliations:** 1 Department of Clinical Neuroscience, Division of Insurance Medicine, Karolinska Institutet, SE-171 77 Stockholm, Sweden; 2 Finnish Institute of Occupational Health, Helsinki, Finland; 3 Center of Epidemiology and Community Medicine, Stockholm County Council, Stockholm, Sweden; 4 The Swedish School of Sport and Health Sciences, Stockholm, Sweden

**Keywords:** Sick leave, Cohort study, Twins, Hospitalization, Longitudinal, Sweden

## Abstract

**Background:**

The associations between a sickness absence spell duration and patient care have been rarely studied. An assumption is that associations would differ by spell duration and by the patient care type, inpatient- or specialized outpatient, due to severity of diseases and/or conditions. We aimed to investigate sickness absence spells in various spell durations as a predictor for subsequent inpatient- and specialized outpatient care separately, and to study if familial confounding plays a role in these associations.

**Methods:**

We followed a population-based sample of Swedish twins born 1925–90 with national registers from 2001 for first incident sickness absence spell (days to calculate spell duration categorized into ≤30 days, 31–90 days, 91–180 days and ≥ 181 days), or no sickness absence, and for inpatient- and specialized outpatient care until 2013 (*n* = 24,975). Cox proportional hazards models were applied for hazard ratios (HR) with 95% confidence intervals (CI) while accounting for covariates and familial confounding.

**Results:**

First incident sickness absence spell across all duration categories was associated with an increased risk of inpatient- (age- and sex adjusted HR 1.28 to 6.05) or specialized outpatient care (HR 1.17–2.50), both in comparison to those without any sickness absence or the shortest sickness absence spell category (1–30 days). The associations remained statistically significant while controlling for covariates or familial confounding.

**Conclusions:**

First incident sickness absence spell increases the risk of inpatient care or specialized outpatient care regardless of the duration of the sickness absence spell. Hence, incident sickness absence spells should be noted and targeted to actions at workplaces as well as in primary and occupational health care.

**Supplementary Information:**

The online version contains supplementary material available at 10.1186/s12913-021-06310-w.

## Background

Sickness absence (SA) is a common practice in health care and the most recent statistics by WHO [1] show that the European Union had on average 12 days of SA per year in 2017 whereas Sweden had 11 days [2]. Consequences of SA such as permanent work incapacity in terms of disability pension, morbidity or mortality [3–5] have merited interest in recent years as the impact is considerable for the individuals, employers and for society. Furthermore, the consequences of SA influence medical (such as hospitalization), psychosocial (comorbidity or related to exclusion from the labour market), but also economic (i.e. loss of income or extra costs) aspects [6–11]. For those being on SA it is always an option to return to work, i.e. SA is targeted to allow an individual to recover and retain work capacity. However, a recent study based on Swedish twins indicated that SA due to mental diagnoses predicted both inpatient and specialized outpatient care and mortality although not accounting for the duration of SA [4]. Until now, relatively few studies have investigated the consequences of various SA spell durations in terms of health care utilization [12, 13] which would be important for public health in terms of preventive actions of increase in need of care.

Although SA spell durations may depend on the underlying condition for seeking care, severity of such condition/illness or on other influential factors such as economics, workplace level factors or else, SA spell duration would be important as it is information that is usually collected and therefore available for interpretations in (occupational) health care [14, 15]. As SA is common, one could consider that early attention to SA could be a trigger or an indicator for initiation of potential preventive means such as workplace or occupational health care interventions.

Genetics is an influential factor that play a role in SA and in many influential factors including age and socioeconomic status, and in consequences of SA (e.g., permanent work incapacity) [4, 16, 17]. Studies have shown familial factors (i.e. genetics and shared, mainly childhood, family environment) to influence the risk of SA, permanent work incapacity but also transitions between them [18–20]. Genetics also play a role in many chronic conditions that usually require healthcare, including high blood pressure [21], low back pain [22], or migraine [23]. Thus, associations between SA duration and health care utilization should preferably be adjusted for familial confounding, an elegant feature provided by twin studies.

In this study based on a population-based sample of Swedish twins with comprehensive coverage of national register data for SA and in- and outpatient care, we hypothesized that the associations between SA and patient care would differ by spell duration in a dose-response manner but also by the patient care type. That is, the associations are expected to be different depending on received inpatient or specialized outpatient care due to severity of diseases and/or conditions.

This study aimed to a) investigate SA in various spell durations as a predictor for subsequent inpatient- and specialized outpatient care and b) to study if familial confounding plays a role in these associations.

## Methods

**Fig. 1:**
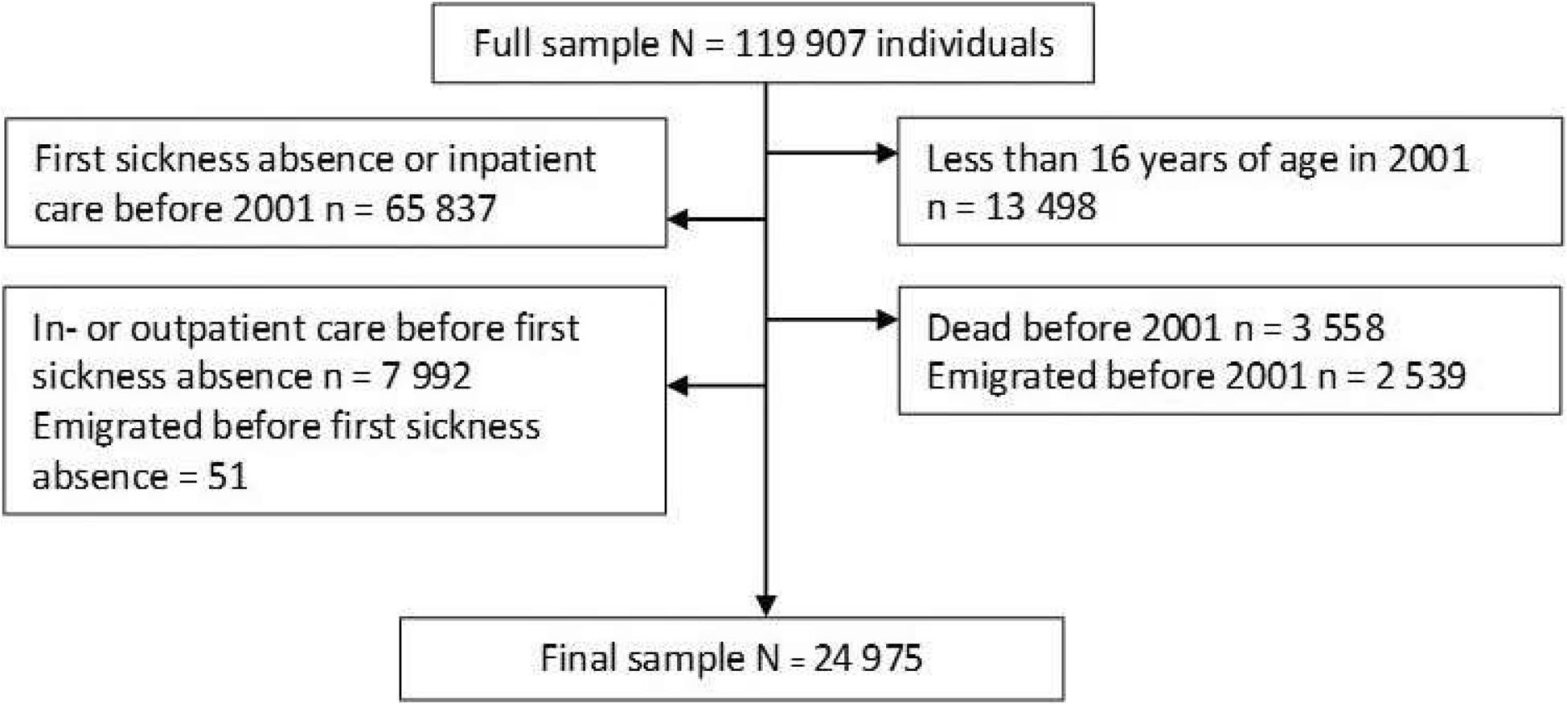
Flow chart of the study sample

This study was based on the data available in the population-based prospective Swedish Twin project Of Disability pension and Sickness absence (STODS) [24]. STODS consists of all twins (*n* = 119,907 individuals) born in Sweden between 1925 and 1990 identified in the Swedish Twin Registry (STR) [24, 25]. Roughly, twins split into one-thirds by being monozygotic (MZ), same-sexed dizygotic (DZ), and opposite-sexed DZ twins (OS) [25]. We limited the STODS data to those alive and living in Sweden in 2001 and at the time not on SA or disability pension, and present (not emigrated or died) during the follow-up. Data on SA and disability pension were from the Micro-Data for Analysis of the Social Insurance System (MiDAS) database from the National Social Insurance Agency. In Sweden, all residents aged 16–65 years and having income from work, unemployment benefits, or student benefits are eligible for the national sickness absence insurance system if they are unable to work due to disease or injury. Furthermore, those unemployed, disability pensioned or retired, can have patient care equally as employed hence we did not account them as censoring. Second, we restricted the sample to those at least 16 years of age and at risk of hospitalization: i.e. not having inpatient- or specialized outpatient care before first incident SA to avoid reverse causation. The inpatient- and specialized outpatient care included dates and diagnoses from the National Board of Health and Welfare. Since we had data for care until the end of 2013, we also restricted all the other data sources until the end of 2013 including emigration (from Statistics Sweden the Longitudinal Integration Database for Health Insurance and Labor Market Studies Register [LISA by Swedish acronym]) [26] and deaths that were censored. Date of death was available from the causes of death register from the National Board of Health and Welfare. Hence, the final study sample included 24,975 individuals (Fig. 1).

Furthermore, we accounted for the fact that our sample included International Classification of Diseases 10th Revision codes (ICD-codes) O00-O99: Pregnancy, childbirth and the puerperium both for SA and inpatient and specialized outpatient care. As pregnancy and childbirth are not considered as illnesses and many will have SA and inpatient or specialized outpatient care during that time, we excluded them from the analyses. However, the number of those with SA due to O00-O99 was 47 since we had diagnosis of SA only from 2005 onwards, i.e. not from the baseline year 2001 as other data in this study. Inpatient or outpatient care due to O00-O99 for 1557 individuals were included in the analyses for censoring reasons. In the final sample, the number of complete twin pairs was 1202 MZ, 1220 DZ, and 672 OS twin pairs. Mean age at baseline was 44.2 years (range 17–76, SD 17.5) and 38% of the final sample were women.

### Duration of a sickness absence spell

We used the first incident SA spell after baseline for the number of SA days as our primary exposure of interest during the follow-up (until the end of 2013) and classified the SA spell duration into categories: 1–30 days, 31–90 days, 91–180 days and ≥ 181 days, or no SA.

### Inpatient- and specialized outpatient care episode

The first incident inpatient- and specialized outpatient care episode with main diagnosis code (ICD 10) after first incident SA spell were our study outcomes. The follow-up was from 1.1.2001 until 31.12.2013 and the censoring was date of emigration or death, whichever occurred first. We used the unique ten-digit Swedish identification number for the linkage of data from the national registers.

### Covariates

Data on covariates including age and sex from STR, family situation (i.e. a combination of marital status and children living at home), education and type of living area were available from Statistics Sweden the LISA database in 2001 [26]. We included these covariates due to their known association both with SA [27] and study outcomes [28].

### Statistical analyses

First, we calculated frequencies and proportions to describe the sample. Then we utilized Cox proportional hazards regression models for hazard ratios (HR) with 95% confidence intervals (CI) using first incident inpatient care and specialized outpatient care separately as outcomes. The models were estimated both for categorized SA spell duration using no SA (0 days) and the shortest SA spell duration category (<=30 days) as reference categories. First, the models were calculated adjusting for age and sex while accounting the non-independency within twin pairs by clustering for 95% CIs. Then we added the covariates (education, family status and living area) all at the same time to the model (i.e. full model) to evaluate their influence on point estimates.

We also conducted conditional Cox proportional hazard regression models for discordant twin pairs to investigate the potential confounding by familial factors (i.e. genetics and early shared environment). Conditional Cox models calculates the HRs for same-sex twin pairs discordant for study outcomes; i.e. a twin in a pair had a patient care episode while the co-twin had not during the follow-up. This allows each twin pair to have their own baseline hazard and controls for familial confounding. These conditional models can be interpreted by comparing the results to the models of the whole cohort. If familial confounding plays a role, then the associations should exist in the analyses of the whole cohort but not in the conditional models. On the other hand, no familial confounding is suggested if the association is also found within discordant twin pairs (i.e. conditional models).

As the proportionality of hazards was violated, we estimated Kaplan-Meier survival curves across SA spell durations to assess their differences but also utilized log-rank tests to analyze survival differences. Furthermore, we computed the person-time-at-risk, incidence rate and 25% quartiles for survival time. All statistical analyses were conducted with Stata version 14.2 MP (Stata Corporation, College Station, TX, USA).

The study was approved by the Regional Ethical Review Board in Stockholm.

## Results

In the final sample (*n* = 24,975), 3943 incident SA spells (16%) took place during the follow-up. Those with SA had more often inpatient care *n* = 2041 (52%) compared to those without SA (*n* = 7901, 38%). The respective rates were *n* = 3466 (88%) and *n* = 17,279 (82%) for specialized outpatient care episode (Table 1). The mean follow-up time was 4.9 years (range 0–13 years, SD 4.4 years). Note that the 0 in follow-up time denotes 1 day (i.e. any outcome or reason for censoring has occurred at earliest the day after the first incident SA spell). Sociodemographic characteristics were the same across inpatient, specialized outpatient and no care groups, except for age: inpatient care was more frequent among older age groups than among those with specialized outpatient care or no care (Table 1). The main ICD-10 diagnoses for inpatient care were O00-O99 17%, I00-I99 16%, and S00-T98 11%, whereas for outpatient care S00-T98 17%, Z00-Z99 12%, and R00-R99 10% (Supplemental Table 1).

**Table 1:** Descriptive characteristics across inpatient and specialized outpatient care episodes vs. no care (i.e. no inpatient or specialized outpatient care)

	Inpatient care (*n* = 8385)		Outpatient care (*n* = 20,743)		No care (*n* = 3901)	
	n	%	n	%	n	%
Categorized SA spell duration*						
0 days	6723	80	17,279	83	3493	90
≤ 30 days	859	10	2006	10	284	7
31–90 days	360	4	718	3	69	2
91–180 days	155	2	295	1	26	1
≥ 181 days	288	3	447	2	29	1
Sex (women)	3446	41	8309	40	1025	26
Age						
16–24	755	9	3765	18	742	19
25–34	667	8	3352	16	887	23
35–44	705	8	2619	13	782	21
44–54	1190	14	3359	16	768	20
55–64	2143	26	3920	19	465	12
≥ 65 years	2894	35	3657	18	163	4
Education						
Low (≤9 years)	2994	36	5637	27	890	23
Intermediate (10–12 years)	3020	36	8419	41	1776	47
High (≥13 years)	1846	22	6026	29	1098	29
Family situation						
Married or cohabitant without children	3502	42	6180	30	623	16
Married or cohabitant with children	864	10	3126	15	803	21
Single without children	3930	47	11,157	54	2349	62
Single with children	58	1	209	1	32	1
Type of living area						
Big cities	2752	33	7610	37	1221	32
Medium-sized cities	2931	35	7339	36	1398	37
Rural areas	2671	32	5723	28	1188	31

*SA sickness absence

All categories of duration of a SA spell predicted both inpatient and specialized outpatient care (analyzed separately) and the covariates played a minor role (Table 2). Furthermore, we cannot rule out the effect of familial confounding since the estimates changed when reference category was those with ≤30 days of SA spell duration, but number of discordant twin pairs were low. While comparing those with SA across various spell durations with those without any SA, the results indicate that SA spell in any duration is a very strong predictor for both inpatient and specialized outpatient care (Table 2). The HRs for SA spell duration categories where only slightly higher in longer duration categories hence indicating no trend of dose-response.

**Table 2:** Cox proportional hazards regressions (HR) with 95% confidence intervals (CI) for associations between SA spell duration and inpatient or specialized outpatient care episodes analyzed separately

categorized SA spell duration	Inpatient care (*n* = 8385)						Outpatient care (*n* = 20,743)					
	Age and sex adjusted model		Full model^a^		Discordant twin pairs (*n* = 711)		Age and sex adjusted model		Full model^a^		Discordant twin pairs (*n* = 603)	
	HR	95%CI	HR	95%CI	HR	95%CI	HR	95%CI	HR	95%CI	HR	95%CI
≤ 30 days	1	ref	1	ref	1	ref	1	ref	1	ref	1	ref
31–90 days	**1.28**	**1.09, 1.50**	**1.28**	**1.09, 1.49**	0.67	0.11, 2.99	**1.17**	**1.06, 1.30**	**1.17**	**1.05, 1.29**	1.03	0.35, 2.98
91–180 days	**1.41**	**1.12, 1.77**	**1.39**	**1.10, 1.74**	2.86	0.14, 58.16	**1.34**	**1.15, 1.57**	**1.34**	**1.14, 1.56**	1.19	0.30, 4.75
≥ 181 days	**1.70**	**1.44, 2.00**	**1.67**	**1.41, 1.98**	1.70	0.14, 21.18	**1.35**	**1.19, 1.52**	**1.35**	**1.20, 1.53**	0.79	0.17, 3.70
no SA (0 days)	1	ref	1	ref	1	ref	1	ref	1	ref	1	ref
≤ 30 days	**2.75**	**2.39, 3.16**	**1.66**	**1.52, 1.81**	**3.18**	**1.84, 5.50**	**1.96**	**1.82, 2.12**	**2.04**	**1.89, 2.21**	**2.39**	**1.78, 3.20**
31–90 days	**3.38**	**2.65, 4.31**	**2.15**	**1.84, 2.52**	**4.93**	**1.76, 13.82**	**2.29**	**1.97, 2.68**	**2.35**	**2.01, 2.75**	**2.49**	**1.44, 4.32**
91–180 days	**4.41**	**3.03, 6.43**	**2.40**	**1.86, 3.10**	**4.90**	**1.19, 20.21**	**3.10**	**2.43, 3.91**	**3.11**	**2.43, 3.97**	**2.28**	**1.13, 4.59**
≥ 181 days	**6.05**	**4.40, 8.33**	**3.00**	**2.50, 3.60**	**5.76**	**1.79, 18.46**	**2.50**	**1.95, 3.20**	**2.60**	**2.03, 3.33**	**3.23**	**1.60, 6.51**

^a^Full model adjusted for age, sex, education, family status and living area

**Fig. 2:**
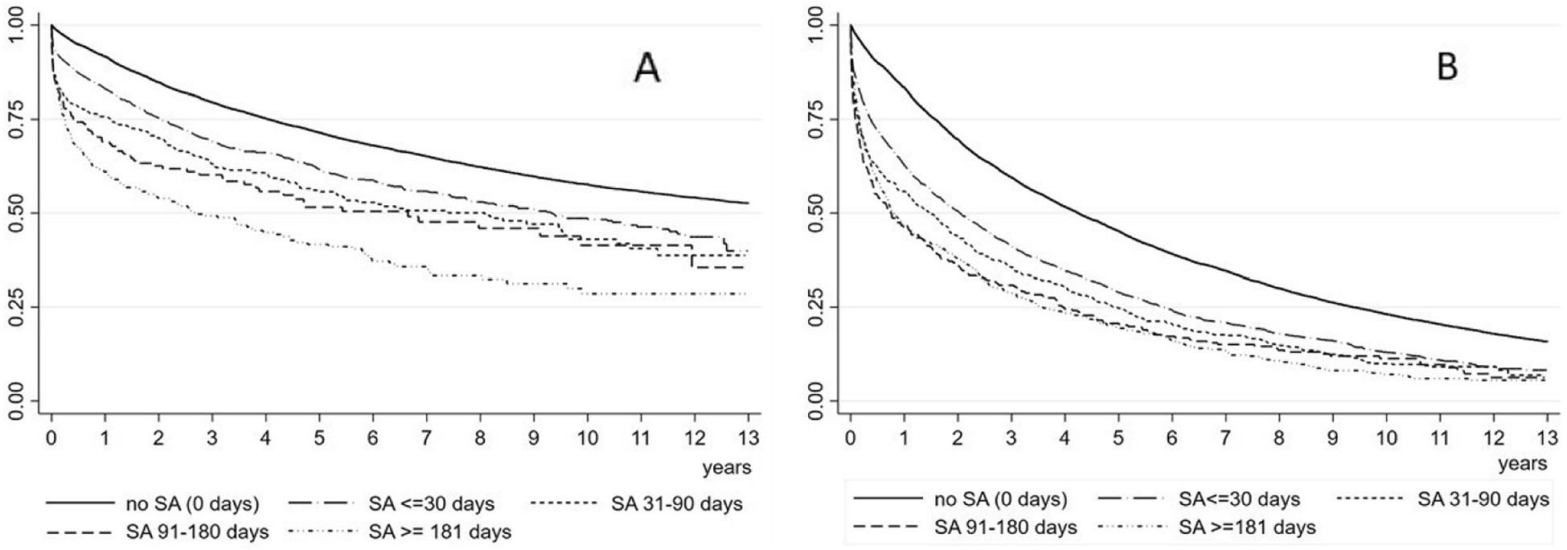
Kaplan-Meier Survival curves across SA spell duration categories for inpatient care (log-rank test for equality of survivor functions *p* < 0.001, panel **a**) and for specialized outpatient care (log-rank test for equality of survivor functions *p* < 0.001, panel **b**)

Kaplan-Meier survival curves for durations of a SA spell are shown in Fig. 2. The log-rank tests for equality of survival functions were highly significant, *p* < 0.001 both for inpatient and specialized outpatient care indicating no differences between categories of duration of a SA spell. This was further confirmed by incidence rates, although the overall relatively low incidence rates followed the trend towards the higher SA spell duration – the higher incidence rate (Supplemental Table 2).

## Discussion

In this comprehensive register data of almost 25,000 Swedish twins we investigated SA spell durations in association with subsequent inpatient or specialized outpatient care. Although SA research with interest in patient care has been recently conducted [3–5], this might have been among the first studies with a focus on SA spell durations. Our results indicate that there is no difference between SA spell duration categories (i.e. ≤30 days, 31–90 days, 91–180 days and ≥ 181 days, or no SA) and survival with inpatient or specialized outpatient care. This finding confirms the earlier results of the existing link between SA and morbidity in terms of patient care [3, 4] but adds to the literature by a similar role for increased risk of inpatient- and specialized outpatient care across all five SA spell duration categories.

Studies of SA and morbidity in terms of patient care have been relatively rare which may reflect the fact that health care utilizations often are followed from onset of a disease, symptom or medication that has required medical attention in a care unit [29, 30]. Alternatively, studies of consequences of SA have focused on mortality, including suicide [3, 9, 11]. Our approach from SA to consequences in terms of patient care stemmed from the hypotheses that associations between a SA spell and patient care would differ by spell duration (i.e. towards an assumption of dose-response effect) but also by the patient care type i.e. inpatient vs. specialized outpatient care (i.e. type and severity of disease assumption). Our survival curves showed no differences across SA spell durations for inpatient nor for specialized outpatient care indicating no support for the hypotheses. The finding of no differences of spell durations is in line with earlier studies in Sweden for suicide or morbidity [3, 12]. However, our results might indicate a need to investigate diagnosis-specific patient care to shed further light on the hypotheses. From a practical point of view considering working life or occupational health care this emphasizes the role of SA regardless of duration as an indicator of compromised health and work ability. Therefore, special attention with relevant actions for prevention should be paid following incidence of SA to avoid any consequences, but patient care in specific.

A worth noting finding of this study relates to the different results depending on the reference categories, that is, “no SA” respectively “short SA” (≤30 days). Specifically, the risk estimates were higher for both inpatient and specialized outpatient care when compared to those without SA than in comparison to short SA. This may imply an overall effect of SA, i.e. the underlying health condition that has earlier been shown to play a role in the prediction of morbidity or mortality [6–11]. Underlying health conditions may also be important due to fact we were not able to rule out the effect of familial confounding in comparison to those with short SA whereas the comparison to no SA indicated no effect of familial confounding. This may reflect the known effect of genetics on SA and predictive factors [4, 16, 17], and in many chronic conditions [21–23].

This study comprising of population-based nationwide data of Swedish twins had several strengths. The coverage of national registries for SA, inpatient- and specialized outpatient care, emigration and deaths were without recall bias and drop-out. Furthermore, our data had relatively long follow-up since 2001 until 2013 enabling to detect both relatively large sample of incident SA spells and patient care episodes. The access to twin data provides possibility to control and assess familial confounding that adds to the population-based studies without twin pair identification. The role of genetics in the associations between SA spell durations and patient care was expected based on earlier studies of SA, patient care and chronic conditions [17–19, 21–23]. These previous findings together with our findings point towards the fact that early attention to and prevention of SA and the underlying conditions would be of importance and that such actions should be initiated already early in the life course to retain health and work capacity.

No studies are without weaknesses. This study may have limited generalizability as it relies on relatively wealthy welfare systems in Sweden but should apply to countries with similar social security and health care such as other Nordic countries. Furthermore, in the literature, very many different categorizations of SA spell durations exist. We used the one used in earlier studies based on Swedish data [3] as that applies to national regulations and would be useful in other settings as well, however, comparisons across studies are limited. Furthermore, we did not account for the diagnosis for SA as that became available in the registers later than our baseline in 2001 and would merit a study of its own. One may also speculate that lack of assessment of diagnosis-specific inpatient- and specialized outpatient care may have flawed the results. However, that would merit another study with relevant hypotheses, but our results indicate also that even larger sample size would be needed to investigate inpatient- or specialized outpatient care for main diagnosis categories.

## Conclusions

Incident SA increases the risk of inpatient care or specialized outpatient care regardless of the duration of the SA spell. The risk for future patient care remains while accounting for various influential factors including familial confounding. Hence, a first SA spell should be noted and targeted by actions at workplaces, and in primary or occupational health care.

## Supplementary Information

**Additional file 1: Supplement Table 1** Frequencies of diagnoses of inpatient and specialized outpatient care episodes among individuals with and without sickness absence (SA). **Supplemental Table 2** Summary of time at risk and incidence rates with quartiles of survival time for inpatient and specialized outpatient care across SA spell duration categories.

## Data Availability

The data cannot be made publicly available. According to the General Data Protection Regulation, The Swedish law SFS 2018:218, The Swedish Data Protection Act, the Swedish Ethical Review Act, and the Public Access to Information and Secrecy Act, these type of sensitive data can only be made available after legal review, for researchers who meet the criteria for access to this type of sensitive and confidential data. Readers may contact the last author regarding these details.

## Abbreviations

CI: Confidence intervals
DZ: Dizygotic
HR: Hazard ratio
ICD-codes: International Classification of Diseases 10th Revision codes
LISA: Statistics Sweden the Longitudinal Integration Database for Health Insurance and Labor Market Studies Register
MiDAS: Micro-Data for Analysis of the Social Insurance System
MZ: Monozygotic
OS: Opposite-sexed
SA: Sickness absence
SD: Standard deviation
STODS: Swedish Twin project Of Disability pension and Sickness absence
STR: Swedish Twin Register
