# Supplementary material for: The predictive role of sickness absence spell durations in associations with inpatient- and specialized outpatient care among a population-based Swedish twin sample
Source: BMC Health Serv Res. 2021 Apr 7;21:315. doi: 10.1186/s12913-021-06310-w (PMC8028110; doi:10.1186/s12913-021-06310-w)
Supplement: Supplementary file 1 — Additional file 1: Supplement Table 1 Frequencies of diagnoses of inpatient and specialized outpatient care episodes among individuals with and without sickness absence (SA). Supplemental Table 2 Summary of time at risk and incidence rates with quartiles of survival time for inpatient and specialized outpatient care across SA spell duration categories. [file 12913_2021_6310_MOESM1_ESM.docx]

**The predictive role of sickness absence spell durations in associations with inpatient- and specialized outpatient care among a population-based Swedish twin sample**

**Authors:** Annina Ropponen^1,2^, Mo Wang^1^, Jurgita Narusyte^1,3^, Sanna Kärkkäinen^1^, Victoria Blom^1,4^, Pia Svedberg^1^

**Supplement Table 1** Frequencies of diagnoses of inpatient and specialized outpatient care episodes among individuals with and without sickness absence (SA)

|  | SA | | | | No SA | | | |
| --- | --- | --- | --- | --- | --- | --- | --- | --- |
| ICD-10 main categories for in- and outpatient care | Inpatient care (n = 2041) | | Outpatient care (n= 3466) | | Inpatient care (n = 7901) | | Outpatient care (n=17279) | |
|  | **n** | **%** | **n** | **%** | **n** | **%** | **n** | **%** |
| A0-A99, B0-B99: Certain infectious and parasitic diseased | 39 | 2 | 45 | 2 | 142 | 2 | 245 | 2 |
| C00-D48, Neoplasms | 163 | 8 | 132 | 5 | 651 | 9 | 665 | 5 |
| D50-D89: Diseases of the blood and blood-forming organs | 11 | 1 | 4 | 0 | 37 | 1 | 23 | 0 |
| E00-E90: Endocrine, nutritional and metabolic diseases | 26 | 1 | 31 | 1 | 72 | 1 | 174 | 1 |
| F00-F99: Mental and behavioural disorders | 96 | 5 | 91 | 3 | 177 | 2 | 132 | 1 |
| G00-G99: Diseases of the nervous system | 41 | 2 | 74 | 3 | 189 | 3 | 170 | 1 |
| H00-H59: Diseases of the eye and adnexa | 14 | 1 | 98 | 4 | 77 | 1 | 1232 | 10 |
| H60-H95: Diseases of the ear and mastoid process | 14 | 1 | 50 | 2 | 58 | 1 | 361 | 3 |
| I00-I99: Diseases of the circulatory system | 247 | 13 | 149 | 6 | 1207 | 17 | 605 | 5 |
| J00-J99: Diseases of the respiratory system | 77 | 4 | 72 | 3 | 295 | 4 | 363 | 3 |
| K00-K93: Diseases of the digestive system | 142 | 7 | 131 | 5 | 630 | 9 | 639 | 5 |
| L00-L99: Diseases of the skin and subcutaneus tissue | 12 | 1 | 96 | 4 | 32 | 0 | 683 | 6 |
| M00-M99: Diseases of the musculoskeletal system | 158 | 8 | 339 | 13 | 495 | 7 | 987 | 8 |
| N00-N99: Diseases of the genitourinary system | 87 | 4 | 154 | 6 | 355 | 5 | 962 | 8 |
| O00-O99: Pregnancy, childbirth and the puerperium | 379 | 19 | 90 | 3 | 1178 | 16 | 127 | 1 |
| Q00-Q99: Congenital malformations, deformations and chromosomal abnormalities | 2 | 0 | 5 | 0 | 6 | 0 | 14 | 0 |
| R00-R99: Symptoms, signs and abnormal clinical laboratory findings | 157 | 8 | 250 | 9 | 702 | 10 | 1232 | 10 |
| S00-T98: Injury, poisoning and certain other consequences of external causes | 271 | 14 | 549 | 21 | 780 | 11 | 1954 | 16 |
| Z00-Z99: Factors influencing health status and contact with health services | 39 | 2 | 312 | 12 | 137 | 2 | 1528 | 13 |

**Supplemental Table 2** Summary of time at risk and incidence rates with quartiles of survival time for inpatient and specialized outpatient care across SA spell duration categories

| Categorized SA spell duration | **time at risk (person years)** | **number of subjects** | **Inpatient care** | | | | **Outpatient care** | | | |
| --- | --- | --- | --- | --- | --- | --- | --- | --- | --- | --- |
|  |  |  | **incidence rate** | **Survival time** | | | **incidence rate** | **Survival time** | | |
|  |  |  |  | **25%** | **50%** | **75%** |  | **25%** | **50%** | **75%** |
| No SA (0 days) | 113792.15 | 21027 | 0.06 | 4.05 | no | no | 0.15 | 1.58 | 4.24 | 9.39 |
| ≤30 days | 6368.87 | 1956 | 0.10 | 2.04 | 9.32 | no | 0.26 | 0.38 | 2.04 | 5.85 |
| 31-90 days | 1652.66 | 581 | 0.13 | 1.09 | 8.08 | no | 0.30 | 0.14 | 1.48 | 4.95 |
| 91-180 days | 622.83 | 252 | 0.16 | 0.41 | 6.65 | no | 0.36 | 0.08 | 0.75 | 3.95 |
| ≥181 days | 934.75 | 388 | 0.22 | 0.25 | 2.73 | no | 0.38 | 0.16 | 0.81 | 3.59 |
| total for those with SA | 9579.11 | 3177 | 0.12 | 1.33 | 7.73 | no | 0.28 | 0.24 | 1.67 | 5.31 |

no = no observations
